# Adsorption Characteristics of Heavy Metals Pb^2+^ and Zn^2+^ by Magnetic Biochar Obtained from Modified AMD Sludge

**DOI:** 10.3390/toxics11070590

**Published:** 2023-07-06

**Authors:** Xiaoting Long, Ruixue Zhang, Rong Rong, Pan Wu, Shiwan Chen, Jipei Ao, Li An, Yuran Fu, Huanhuan Xie

**Affiliations:** 1 College of Resources and Environmental Engineering, Guizhou University, Guiyang 550025, China; lxt15761465127@163.com (X.L.); m19924639193@163.com (R.R.); pwu@gzu.edu.cn (P.W.); swchen@gzu.edu.cn (S.C.); jipeiao@163.com (J.A.); al85369375095678@163.com (L.A.); fuyuran0923@163.com (Y.F.); 2 Key Laboratory of Karst Georesources and Environment, Guizhou University, Ministry of Education, Guiyang 550025, China; 3 Guizhou Karst Environmental Ecosystems Observation and Research Station, Ministry of Education, Guiyang 550025, China; 4 Guizhou Geological and Mineral Foundation Engineering Co., Ltd., Guiyang 550081, China; xiecongming3@163.com

**Keywords:** AMD sludge, magnetic biochar, heavy metals, adsorption mechanism

## Abstract

Acid mine drainage (AMD) sludge can be used to prepare adsorbent materials for the removal of heavy metals in water, which is an effective means for its resource utilization. Magnetic modified biochar (MMB), which can be recovered by magnetic separation, was prepared from sludge generated from the carbonate rock neutralization treatment of AMD and rice straw agricultural waste. Unmodified biochar (UMB) was obtained from rice straw and chemically modified and treated by ultraviolet radiation to produce MMB. The Pb^2+^ and Zn^2+^ adsorption capacities of UMB and MMB were investigated. Simultaneously, the materials were characterized by SEM, FTIR, BET, and ZETA. The results showed that the specific surface area (130.89 m^2^·g^−1^) and pore volume (0.22 m^2^·g^−1^) of MMB were significantly increased compared to those of UMB (9.10 m^2^·g^−1^ and 0.05 m^2^·g^−1^, respectively). FTIR images showed that MMB was successfully loaded with Fe_3_O_4_. The adsorption process of Pb^2+^ and Zn^2+^ onto MMB was consistent with the Langmuir adsorption isotherm and second-order kinetic models, with maximum adsorption capacities of 329.65 mg·g^−1^ and 103.67 mg·g^−1^, respectively. In a binary system of Pb^2+^ and Zn^2+^, MMB preferentially binds Pb^2+^. The adsorption efficiencies of MMB reached >80% for Pb^2+^ and Zn^2+^.

## 1. Introduction

Acid mine drainage (AMD) was recently listed as the second largest global problem by the United Nations because the toxic ions in AMD can cause damage to the surrounding environment and endanger human health [1]. AMD is a type of acidic wastewater rich in sulfate and many types of metal ions, and is formed by the oxidation of metal sulfide minerals produced in coal mines and sulfur-rich metal mines during or after mining works [2,3]. The current treatment technologies for AMD can mainly be divided into active and passive types [4,5]. Active treatment technology is used to add alkaline chemicals to AMD to increase its pH such that metal ions form insoluble precipitates that can be removed. This technology is expensive to operate and maintain. Passive treatment technology relies on natural media (mainly carbonate rocks) to treat AMD using physical, chemical, and biological mechanisms. Due to the low cost and easy availability of carbonate rocks, they are widely used as a reaction medium in AMD treatment. However, the use of carbonate rocks to neutralize AMD is prone to producing a large amount of reddish-brown iron oxide precipitates (hereinafter referred to as AMD sludge), which can cause secondary environmental pollution [6]. These precipitates can hinder the treatment. Therefore, AMD sludge must be cleaned to improve the efficiency of the carbonate rocks treatment of AMD. However, the appropriate disposal of the collected AMD sludge and realization of the resource utilization remain unclear.

AMD sludge, with its large specific surface area and high surface activity, can immobilize heavy metals in solution by adsorption or co-precipitation and reduce heavy metal mobility and bioavailability [7,8,9,10,11]. AMD sludge has been used to adsorb phosphate [12], antimony [13], arsenic [14], phosphorus [15], and other chemicals. Studies have found that AMD sludge contains a significant amount of crystalline amorphous iron, aluminum, and calcium-based minerals, which have a noticeable adsorption effect. However, the direct use of AMD sludge to adsorb metals in wastewater is challenging because AMD sludge forms amorphous nano-flocs or colloids in water and it is difficult to separate the metals after adsorption [16]. This problem can be solved by loading AMD sludge onto biochar. Different sludge-based magnetic biochar materials were successfully prepared by Zeng [17] and Ifthikar et al. [18]. These materials can be separated from the solution by magnets after the adsorption experiments. The results of our preliminary experiments revealed that modified materials can be prepared by loading AMD sludge onto biochar and have a good adsorption capacity with respect to certain metals in water.

Biochar is a solid, carbon-rich particle material that is obtained from agricultural and forestry waste under high-temperature and oxygen-limited conditions. Its surface pore structure is rich and contains numerous functional groups [19]. Biochar can effectively reduce the concentration of pollutants through adsorption; however, it carries the risk of the materials being difficult to separate and could cause the secondary pollution of water bodies. Modification of biochar with magnetization not only provides a stronger adsorption capacity but also facilitates separation and recovery [20,21]. Impregnation, liquid-phase precipitation and pyrolysis are the common methods used to prepare magnetic biochar materials [22,23]. According to Qianlan et al., the magnetic modified biochar (MMB) of grapefruit peel prepared by chemical co-precipitation showed a good adsorption capacity for Pb^2+^ (48.74 mmol·g^−1^) with the coexistence of three metals (Pb^2+^, Cu^2+^, and Zn^2+^) [24]. Xu prepared magnetic MnFe_2_O_4_–sludge biochar composites using the hydrothermal method, which also have a good Pb^2+^ adsorption capacity (174.216 mg·g^−1^) [25]. Pb^2+^ adsorption mechanisms of magnetic MnFe_2_O_4_–sludge biochar composites mainly include physical adsorption, electrostatic attraction, co-precipitation, and complexation. The complexation reaction plays the greatest role in the removal of Pb^2+^. Benias et al. prepared water hyacinth magnetic biochar (Fe_2_O_3_-EC) with a high reusability by co-precipitation and pyrolysis [26]. Because the ionic radius of Zn^2+^ (0.71 Å) is smaller than that of Cu^2+^ (0.73 Å), Fe_2_O_3_-EC is more inclined to adsorb Zn^2+^. In addition, the adsorption efficiencies of Cu^2+^ and Zn^2+^ exceeded 80% in all of these studies. Magnetic biochar has a high specific surface area and porous structure [27], which is conducive to the physical adsorption of metal ions. The pH affects the experiment and the zeta potential of the material. When the surface of the material is electronegative, an electrostatic attraction to positively charged metal ions is produced. When hydroxyl and carbonyl groups are present on the surface of the modified material, they undergo ion exchange and complexation reactions with metal ions. Magnetic biochar improves the adsorption performance of metal ions such as Pb [28], Cu [29], and As [30] and is of advantage because of the easy separation and reusability [23,31].

Based on the successful preparation of AMD sludge-modified biochar [32], we used AMD sludge and rice straw as raw materials in this study to prepare MMB using chemical modification and ultraviolet radiation. The properties and structures of the prepared MMB were characterized using SEM, FTIR, BET, and the ZETA method. Subsequently, adsorption experiments were conducted with MMB on wastewater containing heavy metal ions, and the adsorption performance and mechanism of MMB on Pb and Zn were analyzed and studied. Additionally, the reusability of MMB was explored to provide a theoretical basis for the utilization of AMD sludge as a resource.

## 2. Materials and Methods

### 2.1. Preparation of Experimental Materials

#### 2.1.1. Pre-Treatment

Rice straw collected from the paddy fields near Baihua Lake in Guiyang City, Guizhou Province, China, was repeatedly washed with deionized water and dried in an oven at 60 °C to a constant weight, then cut with a pair of scissors and stored in a self-sealing bag. AMD sludge was collected from a wastewater treatment reaction tank at the Shidong Coal Mine in Guiyang City, Guizhou Province, China. AMD sludge was dehydrated in the suction filter, then dried in an oven at 60 °C until it maintained a constant weight. The dried AMD sludge was ground through a 200-mesh sieve and stored in self-sealing bags for later use. Relevant literature confirmed that Fe (26%) was the main constituent element of AMD sludge, and that Si (2.01%) and Ca (0.59%) were at low levels [32].

#### 2.1.2. Preparation Process

**Figure 1:**
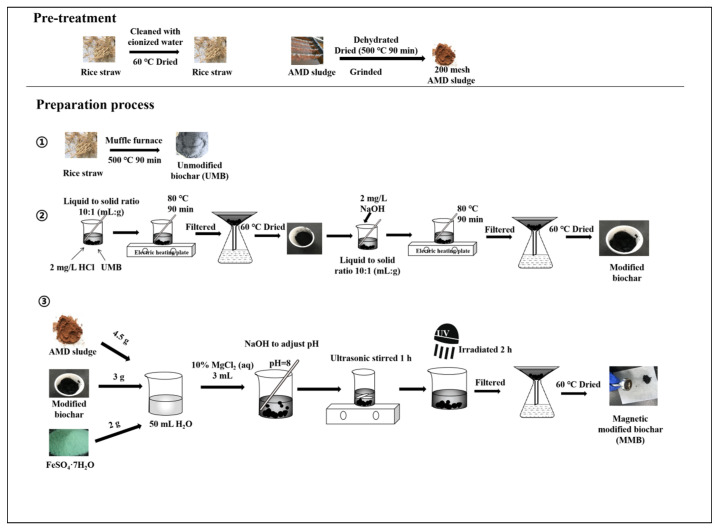
Material preparation flow chart. UV—ultraviolet.

The crushed rice straw was placed into a muffle furnace and carbonized at 500 °C for 90 min to obtain unmodified biochar (UMB), which was ground and passed through a 200-mesh sieve. Then, 2 mg·L^−1^ of HCl solution was mixed with UMB in a liquid:solid ratio of 10:1 (mL:g) in a 250 mL beaker. The mixture was heated at 80 °C for 90 min on a heating plate before being filtered with a separatory funnel after cooling. The filter residue was washed with ultrapure water until the supernatant was neutral and then placed in an oven (60 °C) to dry. The obtained biochar was dried, and the above steps were repeated; however, the HCl solution was replaced with NaOH solution to obtain the modified biochar. Then, 4.5 g of AMD sludge, 2 g of FeSO_4_·7H_2_O, 15 mL of 2 mg·L^−1^ of HCl solution, and approximately 3 g of modified biochar were added to 50 mL of aqueous solution. Then, 3 mL of 10% MgCl_2_ solution was added, and the pH was adjusted to 8 using NaOH solution. The mixture was stirred ultrasonically for 1 h, then irradiated under an ultraviolet lamp for 2 h. The mixture was then filtered, and the filtrate residue was washed with ultrapure water until the supernatant was neutral, then dried in an oven (60 °C) to produce MMB. The preparation procedures are illustrated in Figure 1.

### 2.2. Adsorption Experiments

#### 2.2.1. Adsorption Kinetics

Metal solutions were prepared by dissolving PbNO_3_ and ZnSO_4_·7H_2_O (analytical grade) in deionized water. The adsorption kinetics of the different materials (UMBs and MMBs) were examined by mixing 0.1 g of material, 25 mL of 500 mg·L^−1^ Pb^2+^ or Zn^2+^ experimental solution, and 1 mL of 0.1 mol·L^−1^ NaNO_3_ background electrolyte solution (note: all subsequent experiments were performed under this background electrolyte solution) in a 50 mL centrifuge tube which were then placed in a constant-temperature oscillator and shaken at 25 °C and 200 rpm until sampling. The sampling times were 5, 10, 30, 60, 90, 120, 180, 240, and 300 min. The absorbance of the solution was then measured using an atomic absorption spectrophotometer.

#### 2.2.2. Adsorption Isotherms

Material (0.1 g) was added to a 50 mL centrifuge tube, then 25 mL of different concentrations of Pb^2+^ or Zn^2+^ were added and stirred at a constant speed at 25 °C for 24 h. The samples were then centrifuged and filtered, and the Pb and Zn concentrations in the solutions were determined to study their isothermal adsorption properties separately.

#### 2.2.3. Effect of pH on Adsorption Properties of Materials

A total of 0.1 g of material was added to a series of 50 mL centrifuge tubes, and then different Pb^2+^ or Zn^2+^ concentrations solutions were added. The initial pH of the solution was adjusted from 3 to 7 using 0.1 M HCl or 0.1 M NaOH. The subsequent steps were consistent with those followed in the adsorption isotherm experiment.

#### 2.2.4. Effect of Mixed Ions on Material Adsorption

In this experiment, two systems of Pb–Zn and Zn–Pb were set up for investigation. In the Pb-Zn system, the Pb^2+^ concentration in the solution was unchanged and the Zn^2+^ concentration in the solution gradually increased. In the Zn–Pb system, the Zn^2+^ concentration was unchanged and the Pb^2+^ concentration in the solution gradually increased. The concentration ratios were 1:0.25, 1:0.5, 1:1, 1:1.5, and 1:2. Each solution was mixed with 0.1 g of MMB and then shaken for 3 h in a constant-temperature water bath vibrator (200 rpm, 25 °C). Finally, the supernatant was retrieved to measure the heavy metal concentrations in the solution using an atomic absorption spectrophotometer.

### 2.3. Regeneration Experiments of MMB

#### 2.3.1. Separation Experiments

At pH 7 and 25 °C, a series of concentrations of Pb^2+^ and Zn^2+^ solutions were configured. We added 0.1 g of MMBs into several 50 mL centrifuge tubes, and then added a series of 25 mL Pb^2+^ (or Zn^2+^) solutions at known concentrations at a constant stirring speed and 25 °C for 180 min. The materials were recovered using a magnet after shaking, rinsing, and drying with water repeatedly. Then, the dried materials were added into several 50 mL centrifuge tubes with 25 mL of deionized water. After shaking, the supernatant was taken and the Pb^2+^ and Zn^2+^ concentrations were determined using an atomic absorption spectrophotometer.

#### 2.3.2. Repeated Adsorption Experiments

After adsorption, the MMB was collected via centrifugation. It was then washed with ultrapure water and dried, and directly applied in the next adsorption experiment. The adsorption–regeneration experiments were repeated four times. The experiment conditions were 100 mg·L^−1^ Pb^2+^, 500 mg·L^−1^ Zn^2+^, solid-to-liquid ratio of 4 g·L^−1^, and oscillation for 3 h at 25 °C.

### 2.4. Material Characterization and Sample Testing

A Spectrum 100 infrared spectrometer (PerkinElmer, Waltham, MA, USA) was used to record the infrared spectrum of the materials and analyze their functional group composition. The specific surface area (BET-N_2_ method) and pore size distribution of the materials before and after modification were compared using an ASAP-2020 surface area analyzer (Mike Instruments, Pocatello, ID, USA). The surface structure characteristics of samples under different magnifications before and after modification were observed using an S-570 scanning electron microscope (Hitachi Company, Tokyo, Japan). An atomic absorption spectrophotometer (TAS-990, Beijing Purkinje General Instrument Co., Ltd., Beijing, China) was used to determine the Pb^2+^ and Zn^2+^ concentrations in the solution. DelasaNanoC (McMurritk, NSW, Australia) was used to determine the electro-kinetic potential of the samples.

## 3. Results and Discussion

### 3.1. Adsorption Properties of Pb^2+^ and Zn^2+^

#### 3.1.1. Adsorption Kinetics

**Figure 2:**
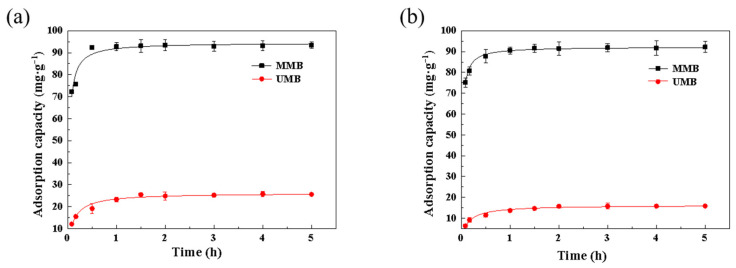
Adsorption kinetics of (**a**) Pb^2+^ and (**b**) Zn^2+^ of the two materials. MMB—magnetic modified biochar, UMB—unmodified biochar.

The Pb^2+^ and Zn^2+^ adsorption kinetics of the two materials are shown in Figure 2. The Pb^2+^ or Zn^2+^ adsorption rates of the two materials were very fast in the first 30 min. As the adsorption sites on the surface of the two materials approached saturation, the adsorption rate decreased and finally reached an equilibrium after 1 h. MMB has a greater Pb^2+^ and Zn^2+^ adsorption capacity, which can be attributed to abundant active sites, that is, the large surface area and pore structure. Factors limiting the adsorption rates include the diffusion of the adsorbent molecules at the interface, electrostatic attraction by the adsorbent surface/repulsion, adsorption potential binding ability of the adsorbent surface, and control of surface chemical reactions [33,34].

The parameters obtained from the adsorption kinetics models (pseudo-first-order, pseudo-second-order, Elovich, and double constant) are shown in Table 1. The correlation coefficients of the four models fitted for Pb^2+^ adsorption by UMB were 0.84, 0.96, 0.93, and 0.88, and those of MMB were 0.75, 0.92, 0.75, and 0.72. The pseudo-second-order model had the highest fit for MMB adsorption of Pb^2+^ or Zn^2+^, which was consistent with the linear form of the already reported pseudo-second-order model [35]. This indicated that the pseudo-second-order model could better describe the kinetic properties of Pb^2+^ adsorption by UMB and MMB. For Zn^2+^ adsorption by UMB and MMB, the correlation coefficients (R^2^) of the pseudo-second-order model were 0.97 and 0.98, respectively, which indicated that the pseudo-second-order model could well describe the adsorption processes of Zn^2+^ by UMB and MMB. The fitting results showed that MMB mainly relied on chemisorption to achieve Pb^2+^ and Zn^2+^ adsorption, and its adsorption rate was mainly controlled by the chemical reaction on the material surface [36,37].

#### 3.1.2. Adsorption Isotherms

**Figure 3:**
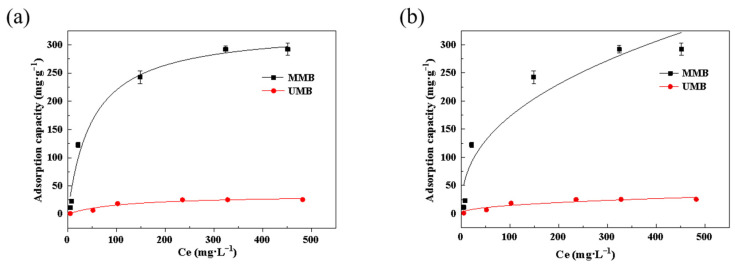
Adsorption isothermal models for Pb^2+^ adsorption by the different materials: (**a**) Langmuir model; (**b**) Freundlich model. Ce: equilibrium mass concentration, mg·L^−1^.

**Figure 4:**
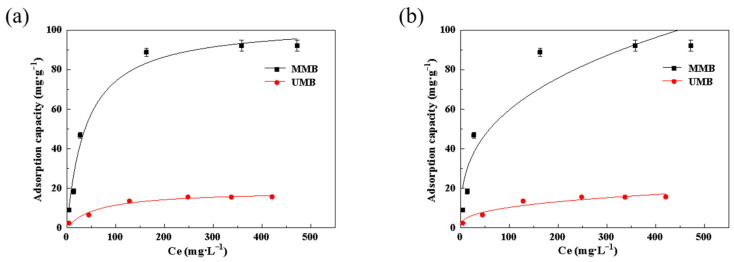
Adsorption isothermal models for Zn^2+^ adsorption by the materials: (**a**) Langmuir model; (**b**) Freundlich model.

The Langmuir model assumes that the molecules adsorbed on the surface have monolayer distributions, the surface has a fixed number of adsorption sites, and that adsorbed molecules do not interact. Figure 3 and Figure 4 and Table 2 and Table 3 demonstrate that the Langmuir model could better describe the process of Pb^2+^ and Zn^2+^ adsorption by MMB. According to the calculated isotherm parameters, the model-calculated values of the equilibrium adsorption of Pb^2+^ by the material before and after modification were 34.11 mg·g^−1^ and 329.65 mg·g^−1^, respectively, which were similar to the measured values of 25.58 mg·g^−1^ and 292.20 mg·g^−1^, respectively. The modeled values of equilibrium adsorption of Zn^2+^ before and after the modification were 18.70 mg·g^−1^ and 103.67 mg·g^−1^, respectively, which were also similar to the measured values of 15.62 mg·g^−1^ and 92.17 mg·g^−1^, respectively. The correlation coefficient (R^2^) for the linear form of Pb^2+^/Zn^2+^ adsorption for both materials was close to 1. This indicated that the MMB surface had many energetically undifferentiated adsorption sites, and that the adsorption process of Pb^2+^ or Zn^2+^ in solution by MMB was more consistent with the Langmuir model [38].

To further evaluate the adsorption effect of the prepared materials on Pb^2+^ and Zn^2+^, the absorption ability and conditions of MMB were compared to those of previously reported adsorbents (Table 4). The results showed that the prepared MMB had strong adsorption effects on Pb^2+^ and Zn^2+^.

#### 3.1.3. Effects of pH and Zeta Potential on Adsorption

**Figure 5:**
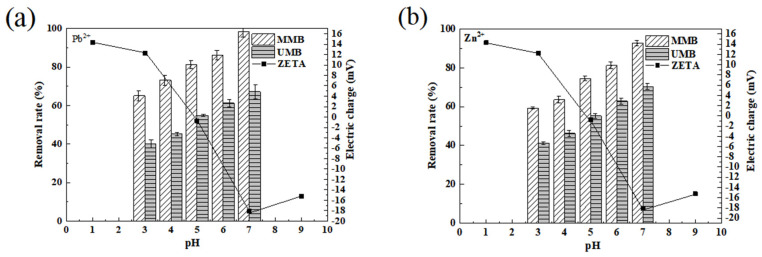
Effect of pH on adsorption of (**a**) Pb^2+^ and (**b**) Zn^2+^ by the materials and ZETA potential analysis.

Changes in the pH of the solution affect the active groups on the surface of materials, leading to changes in their protonation and thus affecting the adsorption process. As Pb^2+^ and Zn^2+^ produce white precipitation under alkaline conditions, the pH range set for this experiment was 3.0–7.0 to avoid interference from precipitation. As seen in Figure 5, the overall increase in solution pH from 3.0 to 7.0 resulted in an increasing trend in Pb^2+^ and Zn^2+^ adsorption on the surface of MMB/UMB. Furthermore, the adsorption capacity of MMB for Pb^2+^ and Zn^2+^ was significantly higher than that of UMB under the same pH conditions. The zeta potential test showed that the electronegativity of MMB was at the maximum (−18.11 mV) at pH 7.0. At this point, the deprotonation of MMB made the surface of MMB negatively charged, which promoted attraction to the positively charged Pb^2+^ and Zn^2+^ through electrostatic interaction, and the maximum adsorption amount was reached.

At a pH below 5.0, the MMB surface became positively charged and electrostatic repulsion occurred. Additionally, the oxygen-containing groups, such as carbonyl and hydroxyl groups, on the surface of MMB easily bound H^+^ in aqueous solution, which then occupied the adsorption sites; the large amount of H^+^ in solution competed with Pb^2+^ (and Zn^2+^) for adsorption, which hindered the migration of Pb^2+^ (Zn^2+^) to the surface of MMB and then decreased the adsorption efficiency. As the pH of the solution increased (5.0–7.0), the amount of H^+^ decreased, and its competing adsorption ability weakened. The increase in dissociation of oxygen-containing groups on the surface of MMB increased the ratio of hydroxyl and carbonyl groups in the form of -COO- and -O- and increased the negative charge of MMB. This enhanced the attraction of MMB to positively charged Pb^2+^ and Zn^2+^ ions.

#### 3.1.4. Effect of Mixed Ions on MMB Adsorption Ability

**Figure 6:**
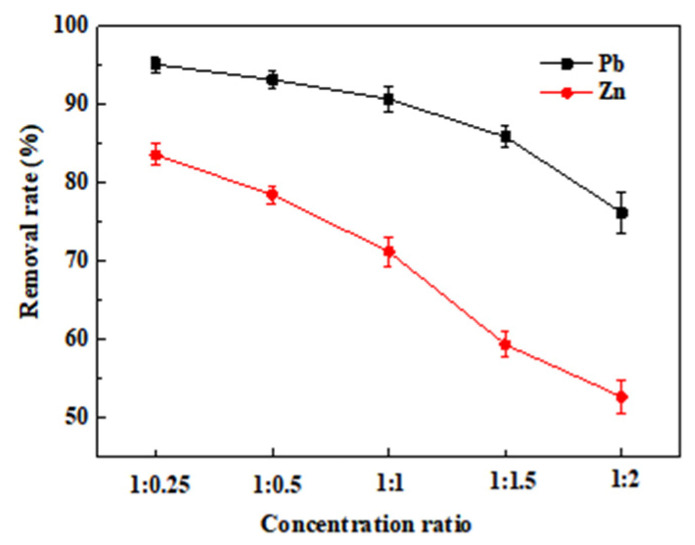
Effect of the binary (Pb and Zn) mixed system on MMB adsorption.

In this experiment, two systems (Pb–Zn and Zn–Pb) were established. In the Pb–Zn system, the Pb^2+^ concentration in the solution was kept constant and the Zn^2+^ concentration in the solution was gradually increased to study the removal of Pb^2+^ by MMB. In the Zn–Pb system, the Zn^2+^ concentration was kept constant and the Pb^2+^ concentration in solution was gradually increased to study the removal of Zn^2+^ by MMB. Figure 6 shows that the adsorption ability of MMB decreases with the increase in the ion concentration due to the addition of competing ions in both systems. As the initial concentration of the interfering ion Zn^2+^ increased from 25 to 150 mg·g^−1^, the decrease in the adsorption ability of MMB for Pb^2+^ was marginal and slow. Furthermore, when the concentration of the interfering ion increased to 200 mg·g^−1^, the Pb^2+^ adsorption by MMB decreased significantly. This indicated that the low Zn^2+^ concentration in the Pb–Zn system had little influence on the ability of MMB to adsorb Pb^2+^ and that high concentrations significantly inhibited Pb^2+^ adsorption by MMB. In the Zn–Pb system, Zn^2+^ adsorption by MMB significantly decreased with the increase in the concentration of interfering ions (Pb^2+^). The results showed that the presence of Pb^2+^ in these systems significantly inhibited the Zn^2+^ adsorption by MMB.

The competitive selective adsorption between ions is related to the hydration radius and electronegativity of heavy metals [46]. The hydration radius of Pb^2+^ (4.01 Å) is smaller than that of Zn^2+^ (4.30 Å), whereas the electronegativity of Pb^2+^ (2.33) is higher than that of Zn^2+^ (1.65) [47]. The radius of hydrated metal ions affects adsorption selectivity, whereby the smaller the radius of hydrated metal ions, the more easily they are adsorbed by the adsorbent [48,49]. Thus, Pb^2+^ was more dominant in competitive adsorption. This also showed that, in addition to the influence of the adsorption mechanism, the properties of individual heavy metal ions also plays an important role in adsorption behavior in competitive systems.

### 3.2. Mechanism of Pb^2+^ and Zn^2+^ Adsorption by the Materials

#### 3.2.1. Microscopic Morphological Changes in UMB and MMB

**Figure 7:**
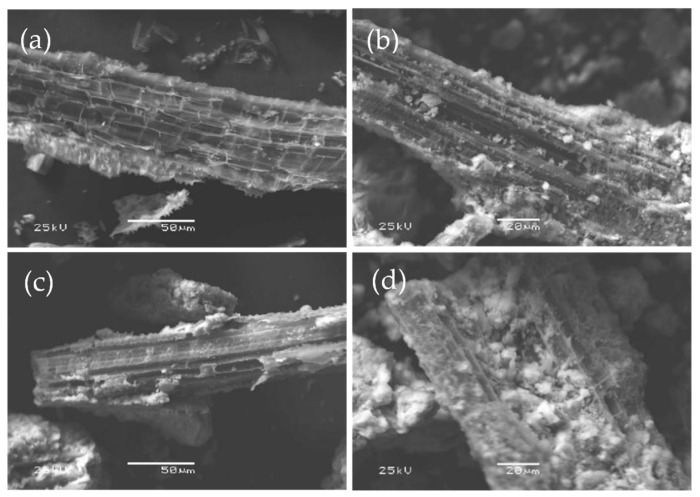
Scanning electron microscopy (SEM) images of UMB and MMB: (**a**) UMB; (**b**) MMB; (**c**) MMB-Pb^2+^; (**d**) MMB-Zn^2+^.

To investigate the changes in the morphological structure of the materials before and after the modification, SEM was performed to study UMB and MMB, and the results are shown in Figure 7. Figure 7a,b show that the surface of UMB was smoother and flatter than that of MMB. After modification, the surface structure of MMB was considerably changed. The surface of MMB became significantly rougher, with more effective adsorption sites and many tiny particles of iron elements attached to the surface (Figure 7d). Figure 7c,d also show that a layer of flocs and many new white flocs [50] (maybe a mixture of Pb^2+^ or Zn^2+^ and iron) were attached to the surface of MMB after the adsorption of Pb^2+^ and Zn^2+^.

#### 3.2.2. Specific Surface Area and Pore Size Distribution of UMB and MMB

**Figure 8:**
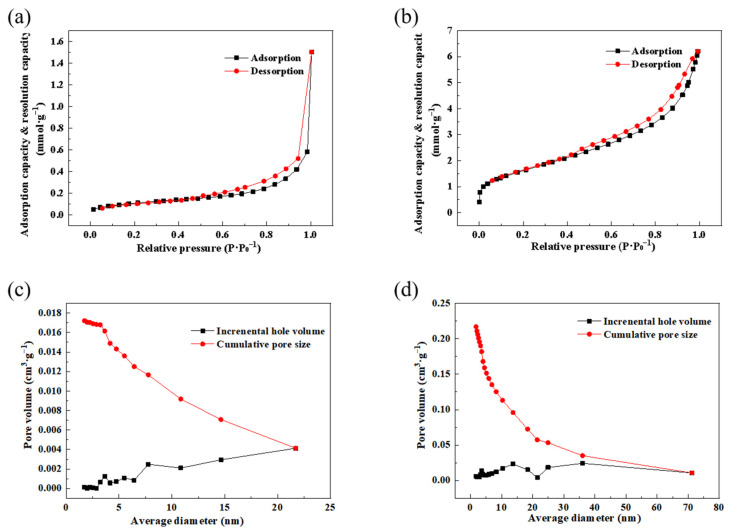
Adsorption and desorption curves and pore size distribution of UMB and MMB: (**a**) UMB adsorption and desorption curves; (**b**) MMB adsorption and desorption curves; (**c**) UMB pore size distribution; (**d**) MMB pore size distribution.

As shown in Figure 8 and Table 5, more fine pores on the surface of MMB were formed following chemical modification, and the specific surface area and pore volume of MMB were enhanced. The measured specific surface area of MMB was 130.89 m^2^·g^−1^, the average pore size was approximately 6.58 nm, and the pore volume was 0.22 cm^3^·g^−1^, which represented a 14.38-fold increase in specific surface area and a 4.4-fold increase in pore volume compared to that of UMB.

#### 3.2.3. FTIR Analysis of MMB before and after Pb^2+^ and Zn^2+^ Adsorption

**Figure 9:**
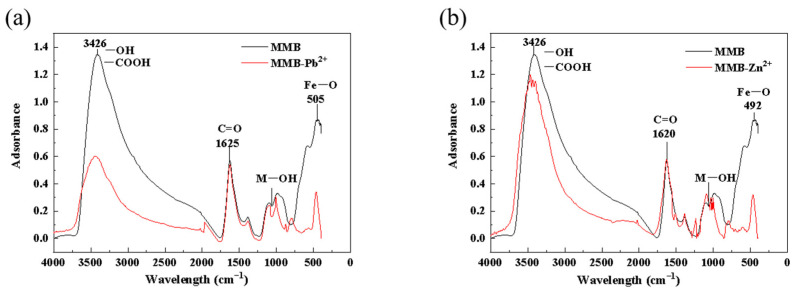
Comparison of infrared spectra before and after adsorption of (**a**) Pb^2+^ and (**b**) Zn^2+^ by MMB.

Figure 9 shows the infrared spectra of MMB before and after Pb^2+^ and Zn^2+^ adsorption. As shown in Figure 9, the peaks at 505 cm^−1^ and 492 cm^−1^ correspond to the vibration of Fe–O bonds [51], which further confirmed the successful loading of Fe_3_O_4_ onto MMB. The increased binding of MMB to -OH in the presence of magnetic iron particles resulted in a broad and extended O-H stretching vibrational peak. A carbonyl (-COOH) vibrational peak occurred at 3426 cm^−1^ [52]. After Pb^2+^ and Zn^2+^ adsorption by MMB, their respective O-H peaks were reduced. It may be that Pb^2+^ (or Zn^2+^) was attracted to the surrounding environment by electrostatic adsorption. Then, some Pb^2+^ (or Zn^2+^) diffused to the surface of MMB, which underwent reactions with H^+^ and the carbonyl group. A previous study [53] confirmed that Pb^2+^ adsorption by magnetic biomass carbon was accompanied by the ionic exchange of Pb^2+^ with H^+^ in O-H and complexation with oxygen-containing functional groups, such as -OH and -COOH, on the surface of the magnetic biomass carbon. C=O was shown at 1620 cm^−1^ [54,55], and there was no significant change in the peak before and after adsorption, indicating that the carbon group was not involved in the reaction. The peak at 1097.3 cm^−1^ corresponded to M-OH (M is Fe) in the MMB, which exhibited an -OH bending vibration, which was likely because M-OH dissociated H^+^ and reacted with Pb^2+^ and Zn^2+^ to form surface complexes [56]. The strength of electrostatic interactions is closely related to the pH of the solution [57]. Figure 5 shows the zeta potential of the material adsorbing Pb^2+^ and Zn^2+^ under different pH conditions. It was found that, in a neutral pH environment, a large number of grafted carbonyl groups on the surface of MMB underwent a deprotonation reaction (-COOH group lost H^+^ to form -COO-, -OH lost H^+^ to form -O-), increasing the degree of dissociation of functional groups and the negative charge. This enhanced the coordination bond force and electrostatic adsorption capacity between MMB, Pb^2+^, and Zn^2+^.

**Figure 10:**
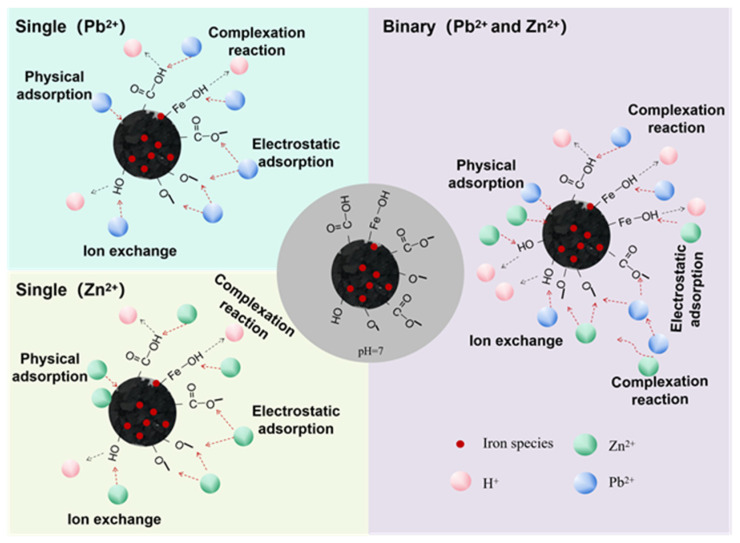
Mechanism diagram of adsorption of heavy metals by MMB.

In summary, the Pb^2+^ and Zn^2+^ adsorption mechanism of MMB mainly involved physical adsorption, ion exchange, electrostatic attraction, and complexation reactions. In the binary system, where Pb^2+^ and Zn^2+^ coexisted, Pb^2+^ and Zn^2+^ competed for the adsorption sites on the surface of MMB, and MMB preferentially bound to Pb^2+^. The principle diagram of the adsorption mechanism of MMB of Pb^2+^ and Zn^2+^ is shown in Figure 10.

### 3.3. Experimental Analysis of Desorption and Regenerative Adsorption by MMB

**Figure 11:**
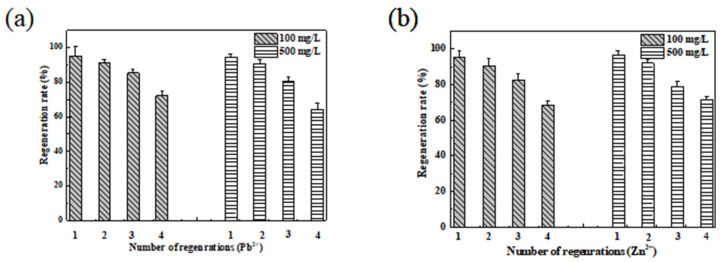
Reusability experiments of MMB adsorption of (**a**) Pb^2+^ and (**b**) Zn^2+^.

**Figure 12:**
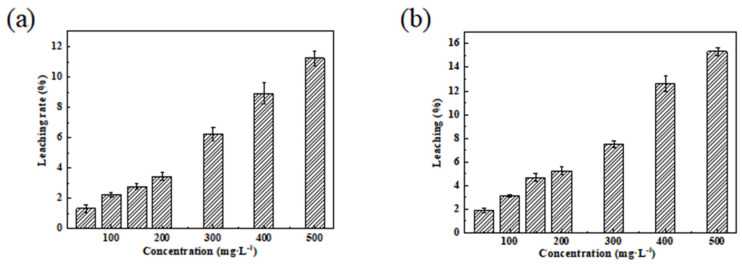
Leaching experiments of MMB for (**a**) Pb^2+^ and (**b**) Zn^2+^.

We analyzed the reuse characteristics of MMB and the recovery of heavy metal resources by studying the cyclic adsorption of MMB. The experiments involving the adsorption–desorption cycling of Pb^2+^ and Zn^2+^ ions by MMB are shown in Figure 11 and Figure 12, and Table 6.

From Figure 11 and Table 6, the Pb^2+^ and Zn^2+^ adsorption process of MMB was reversible, and the regenerative adsorption performance was high. After four cycles of adsorption–regeneration–resorption in 100 mg·L^−1^ Pb^2+^ and Zn^2+^ solutions, the Pb^2+^ removal rate decreased from 95.23% to 72.23% and that of Zn^2+^ decreased from 95.27% to 68.29%; however, the removal rate remained >80% in all three experiments. For the 500 mg·L^−1^ Pb^2+^ and Zn^2+^ solutions, the Pb^2+^ removal rate from MMB decreased from 94.42% to 64.34%, and that of Zn^2+^ decreased from 96.78% to 71.41% after four cycles. The decrease in removal efficiency may have been attributed to the blocking of the pore structure and a decrease in the number of binding sites [58].

The leaching experiment of MMB after Pb^2+^ and Zn^2+^ adsorption was conducted using deionized water as the leaching solution (Figure 12). The results showed that the leaching rate of MMB increased with an increase in the initial heavy metal concentration in solution, and that the leaching rate of MMB was maintained at <5% when the concentration was <100 mg·L^−1^. Thus, MMB had good adsorption stability for low Pb^2+^ and Zn^2+^ concentrations in solutions.

Under the applied magnetic field (the circular surface of the magnet with a 1.5 cm diameter), MMB was rapidly separated from air and water molecules and adsorbed stably on the magnet, which is important for future resource recycling.

## 4. Conclusions

MMB was prepared from modified AMD sludge and rice straw, and the specific surface area was greatly improved compared to UMB and reached approximately 130.89 mg·g^−1^, which was 14.38-times higher than that of UMB. Langmuir and the secondary kinetic model well described the adsorption process of the materials for the two heavy metals, and showed the adsorption of MMB mainly relied on chemisorption and that the process followed monolayer adsorption. The maximum Pb^2+^ and Zn^2+^ adsorption capacities at 25 °C and pH 7 were 329.65 and 103.67 mg·g^−1^, respectively.

In the Pb^2+^ and Zn^2+^ binary system, the maximum adsorption capacity of MMB was lower than that in the single system. MMB had a stronger bond to Pb^2+^ than to Zn^2+^ in this system. Mechanisms underlying the MMB adsorption of heavy metals included physical adsorption, ion exchange, electrostatic attraction, and complexation.

MMB showed good reproducibility, and after three cycles of adsorption–regeneration, the adsorption efficiency of the material for Pb^2+^ and Zn^2+^ reached >80%. Additionally, it did not undergo extensive desorption, and re-released the adsorbed heavy metals into the environment, suggesting that MMB can be a potential, environmentally friendly adsorbent for treating heavy metal contamination.

Although this study contributes to the resource utilization of AMD sludge and provides novel ideas for the removal of heavy metals (Pb^2+^ and Zn^2+^), actual heavy metal-contaminated wastewater has a complex composition. Therefore, the practical application of the MMB material requires further study.

## Figures and Tables

**Table 1:** Kinetic model parameters for Pb^2+^ and Zn^2+^ adsorption by the different materials.

Adsorbent	Pseudo-First-Order			Pseudo-Second-Order			Elovich			Double Constant		
	q/mg·g^−1^	k	R^2^	q/mg·g^−1^	k	R^2^	α	β	R^2^	k	n	R^2^
UMB-Pb^2+^	24.49	6.17	0.84	26.17	0.33	0.96	276.66	0.29	0.93	21.34	0.16	0.88
MMB-Pb^2+^	92.33	15.54	0.75	94.56	0.36	0.92	0.12	0.19	0.75	88.43	0.06	0.72
UMB-Zn^2+^	15.04	5.12	0.87	16.24	0.43	0.97	592.89	0.42	0.95	12.77	0.18	0.90
MMB-Zn^2+^	90.47	19.50	0.77	93.27	0.53	0.98	0.13	0.25	0.86	87.87	0.05	0.84

**Table 2:** Isothermal model parameters for Pb^2+^ adsorption by the materials.

Adsorbent	Langmuir			Freundlich		
	R^2^	Q_max_/mg·g^−1^	K/L·mg^−1^	R^2^	K/mg·g^−1^	n
UMB	0.93	34.11	0.01	0.85	1.95	0.41
MMB	0.98	329.65	0.02	0.90	25.9	0.44

**Table 3:** Isothermal model parameters for adsorption of Zn^2+^ by the materials.

Adsorbent	Langmuir			Freundlich		
	R^2^	Q_max_/mg·g^−1^	K/L·mg^−1^	R^2^	K/mg·g^−1^	n
UMB	0.97	18.7	0.02	0.91	2.01	0.31
MMB	0.98	103.67	0.03	0.87	12.17	0.35

**Table 4:** Comparison of Pb^2+^ and Zn^2+^ adsorption onto different magnetic biochar types. AMD—acid mine drainage. Q_m_—maximum surface adsorption capacity (mg·g^−1^).

Magnetic Biochar	Conditions	Q_m_ (mg·g^−1^)	Reference
Magnetic sludge biochar	pH 6, 298 K	249.00 (Pb)	Ifthikar et al., 2017 [18]
ZnS-modified magnetic biochar	pH 6, 298 K	367.65 (Pb)	Yan et al., 2015 [39]
Magnetic CeO_2_–MoS_2_ hybrid biochar	pH 4, 298 K	263.64 (Pb)	Li et al., 2019b [40]
Surface-modified magnetic biochar	pH 5, 298 K	103.00 (Pb)	Zahedifar et al., 2021 [41]
Silica-coated magnetic nanocomposites	pH 4–6, 298 K	14.90 (Pb)	Nicola et al., 2020 [42]
Iron oxide–silica nanocomposites	pH 4, 298–318 K	6 (Zn)	Ianăşi et al., 2021 [43]
EDTA and chitosan bi-functionalized magnetic bamboo biochar	pH 5, 298 K	50.80 (Zn)	Zhang et al., 2022a [44]
Calcined magnetic biochar made from banana peel	pH 6, 298 K	72.80 (Zn)	Oladipo et al., 2019 [45]
Biochar prepared from sleeve skin	---, 298 K	48.74 (Pb)/10.26 (Zn)	Wu et al., 2021 [24]
Magnetic modified biochar by AMD sludge	pH 7, 298 K	329.65 (Pb)/103.67 (Zn)	This study

**Table 5:** Specific surface area, pore volume and pore size of UMB and MMB.

Adsorbent	Specific Surface Area (m^2^·g^−1^)	Pore Volume (cm^3^·g^−1^)	Aperture (nm)
UMB	9.10	0.05	22.91
MMB	130.89	0.22	6.58

**Table 6:** Four adsorption–desorption cycles of MMB.

Capacity	Cycles	Pb^2+^		Zn^2+^	
		100 (mg·L^−1^)	500 (mg·L^−1^)	100 (mg·L^−1^)	500 (mg·L^−1^)
Adsorption/desorption	1	95.23%	94.42%	95.27%	96.78%
	2	91.21%	90.88%	90.48%	92.14%
	3	85.25%	80.21%	82.69%	78.92%
	4	72.23%	64.34%	68.29%	71.41%

## Data Availability

We have full control of all primary data, and we agree to allow the journal to review our data if requested.
